# Establishment of an *in vivo*-based assay using a silkworm infection model for phenotypic evaluation of antimicrobial drug combinations against *Mycobacterium abscessus*

**DOI:** 10.1128/aac.01665-25

**Published:** 2026-02-19

**Authors:** Akiho Yagi, Motoko Shinohara, Yusuke Minato, Ryuji Uchida

**Affiliations:** 1 Division of Natural Product Chemistry, Faculty of Pharmaceutical Sciences, Tohoku Medical and Pharmaceutical University 34836, Sendai, Miyagi, Japan; 2 Center for Infectious Disease Research, Fujita Health University 12695, Toyoake, Aichi, Japan; City St George's, University of London, London, United Kingdom

**Keywords:** combination therapy, *Mycobacterium abscessus*, *Bombyx mori*, silkworm

## Abstract

*Mycobacterium abscessus* exhibits high intrinsic drug resistance, requiring combination therapy. We developed a silkworm (*Bombyx mori*) infection model as a whole-organism, *in vivo*-based platform for quantitative, outcome-based evaluations of antimicrobial combinations. The system, examined using clarithromycin-amikacin and imipenem-cefoxitin combinations, showed interaction profiles that were qualitatively consistent with those observed *in vitro*. This rapid, reproducible, and ethical assay enables reliable phenotypic assessments of synergistic or antagonistic effects and may facilitate the evaluation and prioritization of antimicrobial combination regimens in preclinical studies.

## INTRODUCTION

Infections caused by nontuberculous mycobacteria (NTM) are increasing worldwide and remain difficult to treat because of their intrinsic resistance to many commonly used antimicrobials (1). Among NTM, *Mycobacterium abscessus* is recognized as the most drug-resistant species, leaving few therapeutic options (1). Current clinical guidelines recommend prolonged multidrug combination therapy (2). Clarithromycin (CLR) resistance further limits available treatments, highlighting the need for additional agents that may be incorporated into combination therapy. Consequently, efforts to identify synergistic drug combinations are ongoing. Many combinations have been evaluated *in vitro*, and several, including dual β-lactams, have been examined *in vivo* (3, 4). Nevertheless, these *in vivo* studies are limited to specific dosing conditions, and comprehensive evaluations of dose-dependent drug interactions have rarely been conducted.

Although mammalian models, such as mice, are commonly used for these evaluations, the establishment of a persistent *M. abscessus* infection in these models is technically difficult and involves considerable cost and ethical concerns (5). Furthermore, it remains unclear whether *in vitro* drug interactions may be reliably reproduced *in vivo*. Therefore, the need for reliable alternative models that efficiently and quantitatively assess drug interactions *in vivo* is increasing. While *in vitro* combination assays are indispensable for analyzing direct antimicrobial interactions, they cannot capture host-dependent therapeutic outcomes. To address this limitation, we employed a silkworm infection model as a whole-organism system to quantitatively evaluate the effects of antimicrobial combinations on survival. This model is intended to complement *in vitro* assays by providing an outcome-based assessment of combination effects within an integrated biological context, rather than to recapitulate mammalian pharmacokinetics.

Alternative *M. abscessus* infection models using *Drosophila melanogaster* (6), zebrafish (7), and *Galleria mellonella* (8) have been reported, and each has its limitations. *Drosophila* and zebrafish cannot be maintained at 37°C, while the small size of *G. mellonella* hinders quantitative evaluations. In contrast, the silkworm infection model with *M. abscessus* can be maintained at 37°C and supports reproducible systemic infections, enabling quantitative assessments of antimicrobial efficacy within a short period (9). This model allows for the ethical testing of numerous individuals and aligns with the 3Rs principle. Furthermore, the silkworm’s small body weight enables evaluation with minimal compound quantities, making it advantageous for screening limited or valuable molecules. Based on these advantages, we selected the silkworm infection model and established a practical assay system for comprehensive, dose-dependent evaluations of antimicrobial combinations.

**Fig 1:**
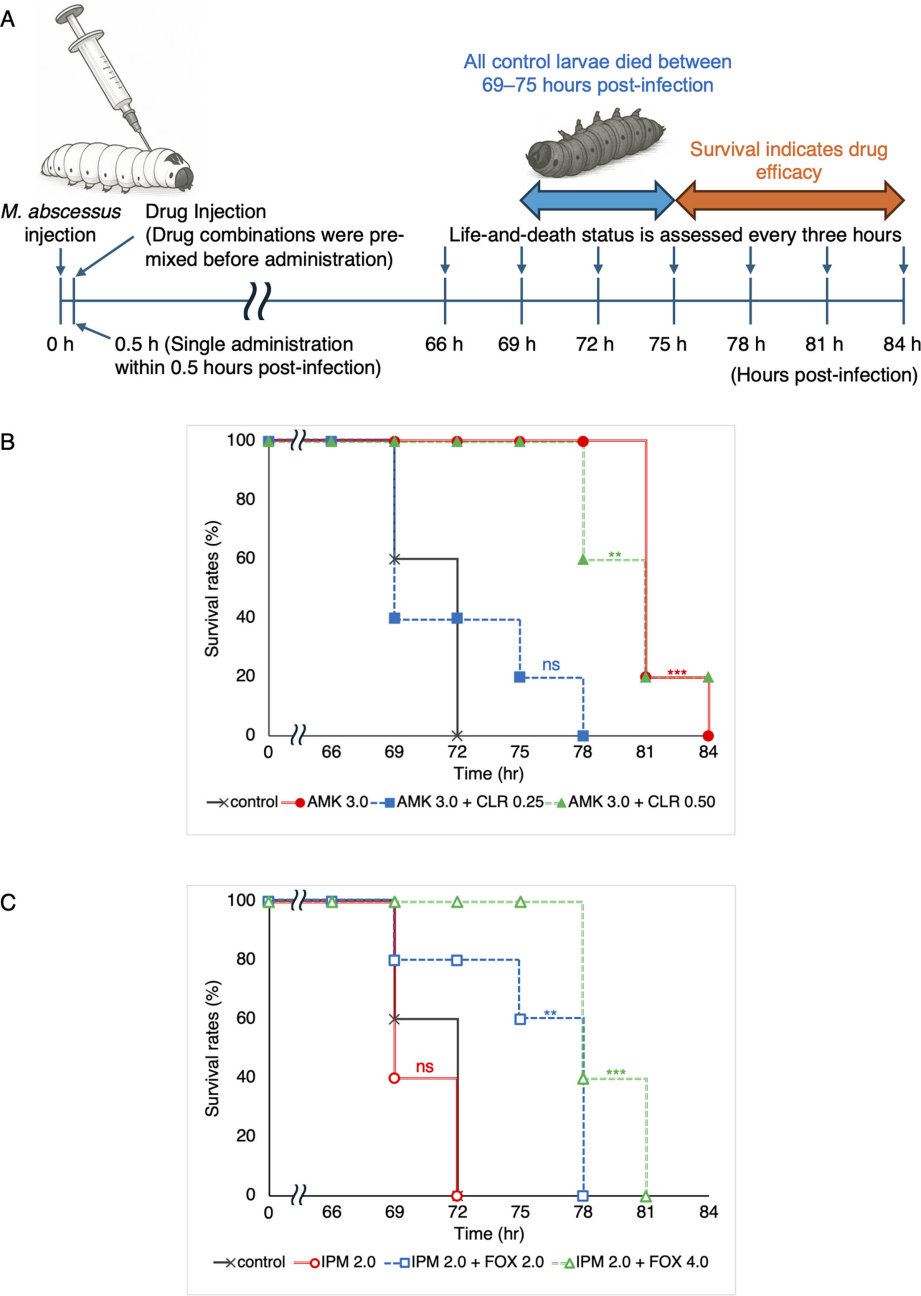
*In vivo* evaluation of drug efficacy using the silkworm infection model with *M. abscessus*. (**A**) Experimental scheme for the infection and treatment protocol. (**B**) Kaplan-Meier survival curves for AMK and CLR. Filled circles, AMK (3.0 µg/larva·g); filled squares, AMK (3.0 µg/larva·g) + CLR (0.25 µg/larva·g); filled triangles, AMK (3 µg/larva·g) + CLR (0.50 µg/larva·g); ×, control. (**C**) Kaplan–Meier survival curves for IPM and FOX. Open circles, IPM (2.0 µg/larva·g); open squares, IPM (2.0 µg/larva·g) + FOX (2.0 µg/larva·g); open triangles, IPM (2.0 µg/larva·g) + FOX (4.0 µg/larva·g); ×, control. Each experiment was performed twice, and reproducible results were obtained. Survival curves were compared with controls using the log-rank test (***P < 0.005, **P < 0.05, ns = not significant).

The efficacies of drug combinations were evaluated using a silkworm infection model with *M. abscessus* (Fig. 1A). Fifth-instar silkworm larvae (2.0 ± 0.1 g, *n* = 5) were infected with *M. abscessus* JCM13569 (ATCC19977) by injecting a bacterial suspension (approximately 2.5 × 10^6^  CFU/mL, 50 µL) into the hemolymph. We previously reported that this infection condition caused 100% mortality within 69–75 h at 37°C (9). To allow for a survival-based evaluation prior to natural death, silkworms were monitored for up to 84 h post-infection. Each drug was administered in a single 50 µL dose, either individually or as a premixed solution, within 30 min after infection. To select the appropriate dose range for the combination study, we initially confirmed that each drug exhibited dose-dependent therapeutic efficacy when administered alone (Table S1 and Fig. S1). Based on these results, five dose levels were selected for each drug, and all possible dose combinations were systematically evaluated. Kaplan-Meier survival curves were generated to assess life-prolonging effects (Fig. 1B and C), and detailed survival data and Kaplan-Meier curves for all combinations are provided in the Supplementary Information (Fig. S1 and Table S1).

The results obtained confirmed that under the CLR and AMK combination, the co-administration of CLR at 0.25 µg/larva·g shortened survival significantly more than AMK alone (Fig. 1B). This antagonistic effect was also observed at lower CLR doses (0.13 and 0.063 µg/larva·g) (Fig. S1 and Table S1). However, no antagonism was observed when CLR was administered at 0.5 µg/larva·g, a dose that was as effective as monotherapy (Fig. 1B). Therefore, the antagonism between CLR and AMK reported *in vitro* (10) was also observed in the silkworm infection model. In contrast, for the IPM and FOX combination, the co-administration of IPM at 2 µg/larva·g with FOX at 2 or 4 µg/larva·g—doses that were individually ineffective—significantly prolonged survival (Fig. 1C). Similar results were observed across other dose levels, and the combination exerted clear synergistic effects *in vivo* (Fig. S1 and Table S1). This result is consistent with previously reported synergy in both *in vitro* studies and mouse models (3, 11), further supporting the validity of our evaluation system. This assay was also independently repeated on different dates and with different dosing lots, yielding reproducible results.

**Fig 2:**
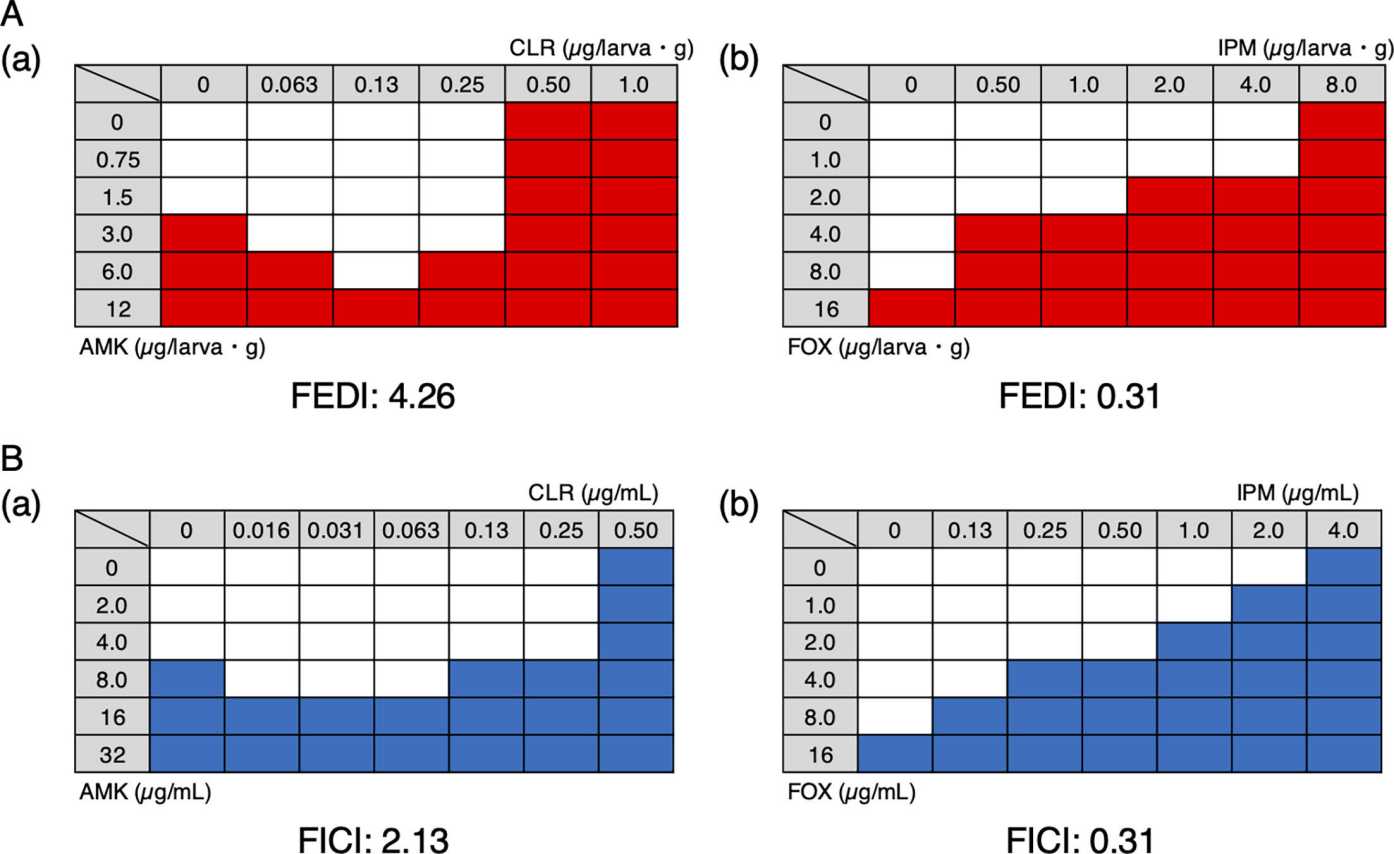
Heat maps of the combinatorial efficacy of clinical drugs against *M. abscessus*. (**A**) (**a**) AMK and CLR, (**b**) IPM and FOX *in vivo* by a silkworm infection model. Red cells indicate wells showing ≥80% larval survival. (**B)** (**a**) AMK and CLR, (**b**) IPM and FOX *in vitro* by a microdilution method. Blue cells indicate wells showing ≥90% inhibition of bacterial growth.


To further enhance the utility of this evaluation system, we established an index for quantitatively assessing drug combination effects *in vivo*. Survival rates at the time when all control larvae (injected without drug treatment) had died were visualized as heatmaps (Fig. 2A), with red cells indicating conditions achieving ≥80% survival, defined as 80% effective doses (ED_80_). Using this matrix, we calculated the fractional effective dose index (FEDI), defined as the sum of the ratios of each drug’s ED_80_ in combination with its ED_80_ as monotherapy.

$$FEDI=\frac{ED_{80},\;Drug\;A\;in\;combo}{ED_{80},Drug\;A\;alone}+\frac{ED_{80},Drug\;B\;in\;combo}{ED_{80},Drug\;B\;alone}$$

This index allows intuitive interpretations, with values <0.5 indicating synergy, 0.5–1 indicating additivity, and >2 indicating antagonism—similar to the fractional inhibitory concentration index (FICI) used *in vitro*. As proof of concept, the CLR and AMK combination yielded FEDI of 4.26, demonstrating clear antagonism, whereas IPM and FOX resulted in FEDI of 0.31, indicating strong synergy. These values corresponded well with *in vitro* FICI values (2.13 and 0.31, respectively) (Fig. 2B), confirming the consistency of this *in vivo* system with established *in vitro* findings. Although optimal dosing ratios were not completely identical between the two systems, this may reflect pharmacokinetic and physiological factors not captured *in vitro*. Therefore, a quantitative *in vivo* evaluation is essential for bridging this gap and designing more clinically relevant combination regimens.

Previous studies have reported that pharmacokinetic parameters and ED₅₀ values obtained in the silkworm model show trends comparable to those observed in mammalian systems (12–14), supporting its practical utility for prioritizing drug combinations prior to mammalian testing. A limitation of the silkworm infection model is that drug exposure under the experimental conditions is relatively static and does not fully recapitulate mammalian pharmacokinetic profiles (15), as is often the case for alternative animal infection models that are not designed to reproduce mammalian pharmacokinetics. In this study, our objective was not to reproduce mammalian pharmacokinetics, but to evaluate drug-drug interactions as phenotypic therapeutic outcomes in a whole-organism infection context. Because single agents and their combinations were assessed under identical exposure conditions, relative changes in survival attributable to drug combinations could be quantitatively compared independent of PK matching.

Thus, while the silkworm model does not reproduce mammalian pharmacokinetics, it serves as a complementary platform for phenotypic evaluation of antimicrobial combinations, particularly in contexts where relative interaction effects rather than precise PK profiles are of interest.

In conclusion, we herein developed an *in vivo*-based assay system using a silkworm infection model for quantitative evaluations of antimicrobial drug combinations. This platform enables reproducible, dose-dependent assessments of synergistic and antagonistic interactions with a high throughput and minimal ethical burden. Thirty-six treatment conditions were evaluated twice for reproducibility, involving 360 silkworms (*n* = 5 per group × 36 groups × 2 experiments). This scale of whole-organism combination testing would be difficult to achieve using mammalian models, underscoring the practical utility of this system. In the future, this platform may be applied to evaluate and prioritize antimicrobial drug combinations prior to translation to mammalian models.

## SUPPLEMENTAL MATERIAL

Supplemental material aac.01665-25-s0001.docx Supplemental methods; Table S1; Fig. S1.

ASM does not own the copyrights to Supplemental Material that may be linked to, or accessed through, an article. The authors have granted ASM a non-exclusive, world-wide license to publish the Supplemental Material files. Please contact the corresponding author directly for reuse.
