# Supplementary material for: Establishment of an in vivo-based assay using a silkworm infection model for phenotypic evaluation of antimicrobial drug combinations against Mycobacterium abscessus
Source: Antimicrob Agents Chemother. 2026 Feb 19;70(4):e01665-25. doi: 10.1128/aac.01665-25 (PMC13041385; doi:10.1128/aac.01665-25)
Supplement: Supplemental material — Supplemental methods; Table S1; Fig. S1. [file aac.01665-25-s0001.docx]

**Supplementary Information**

**Establishment of an *In Vivo*-based Assay Using a Silkworm Infection Model for Phenotypic Evaluation of Antimicrobial Drug Combinations against *Mycobacterium abscessus***

Akiho Yagi, Motoko Shinohara, Yusuke Minato, Ryuji Uchida

Experimental……………………………………………………………………………2

Table S1 Survival rates (%) of *M. abscessus*-infected silkworms under individual and combinatorial drug treatment conditions………………………………………………5

Figure S1 Kaplan-Meier survival curves of *M. abscessus*-infected silkworms under individual and combinatorial drug treatment conditions………………………………6

**Experimental**

**Materials**

Amikacin (AMK) sulfate, imipenem (IPM) monohydrate, bovine serum albumin, and polyoxyethylene (20) sorbitan monolaurate were purchased from FUJIFILM Wako Pure Chemical Industries (Osaka, Japan). Clarithromycin (CLR) and cefoxitin (FOX) sodium were obtained from Tokyo Chemical Industries (Tokyo, Japan). Middlebrook 7H9 broth was supplied by Becton, Dickinson and Company (Franklin Lakes, NJ, USA). Glucose and NaCl were purchased from Nacalai Tesque, Inc. (Kyoto, Japan). All other chemicals used in this study were of special grade.

**Microorganisms**

*Mycobacterium abscessus* JCM 13569 was used in the microdilution assay and the silkworm infection assay. Strain JCM 13569 was provided by the Japan Collection of Microorganisms, RIKEN BRC, which is participating in the National BioResource Project of MEXT, Japan.

**Assay for anti-*M. abscessus* activity**

***In vitro* broth microdilution method**

The broth microdilution method was performed according to a previously established method [1]. *M. abscessus* was grown for 7 days at 37 °C in Middlebrook 7H9 broth (Middlebrook 7H9 broth 1.04%, polyoxyethylene (20) sorbitan monolaurate 0.05%, bovine serum albumin 0.5%, glucose 0.2%, and NaCl 0.085%) up to approximately 1.0 × 10^9^ CFU/mL. Culture suspension was then diluted 500 times with the same fresh broth. The suspension (95 μL) was added to each well of a 96-well microplate with or without test samples (5 μL in MeOH). The microplate was incubated at 37 °C for 5 days. Turbidity was visually assessed, and the MIC value was defined as the lowest concentration of the test compounds at which bacterial growth was inhibited by 90% of control growth (no compound).

***In vivo*-mimic silkworm infection model**

The silkworm infection model was performed according to a previously established method [1]. Fertilized silkworm eggs of *Bombyx mori* (Hu·Yo × Tukuba·Ne) were purchased from Ehime Sansyu (Ehime, Japan) and reared on an artificial diet (Silk Mate 2M; Nihon Nosan Kogyo, Kanagawa, Japan) in an incubator at 27 ºC until the fourth molting stage. On the first day of the fifth-instar larval stage following molting, silkworms were fed the artificial diet until their body weight reached approximately 2 g. On the second day, the *M. abscessus* suspension (6.25 × 10^4^ CFU/larva·g in 50 µL Middlebrook 7H9 broth) was injected into the hemolymph (n = 5) using a disposable 1-mL syringe (NIPRO, Osaka, Japan) with a 27-gauge needle (TERUMO, Tokyo, Japan). Within 30 minutes, 50 µL of the test compound (in saline or 10% DMSO) was administered. Infected silkworms were maintained without feeding at 37 °C, and survival was monitored for 66 to 84 hours post-injection. The ED_80_ value was defined as the dose required to achieve 80% survival, normalized to per gram of body weight.

**References**

1. Hosoda K, Koyama N, Hamamoto H, Yagi A, Uchida R, Kanamoto A, Tomoda H. 2020. Evaluation of anti-mycobacterial compounds in a silkworm infection model with *Mycobacteroides abscessus*. Molecules 25:4971.

**Table S1 Survival rates (%) of *M. abscessus*-infected silkworms under individual and combinatorial drug treatment conditions**

**Figure S1 Kaplan-Meier survival curves of *M. abscessus*-infected silkworms under individual and combinatorial drug treatment conditions**

**Figure S1 (continued)**
